# Big Data in Medicine, the Present and Hopefully the Future

**DOI:** 10.3389/fmed.2019.00263

**Published:** 2019-11-15

**Authors:** Michela Riba, Cinzia Sala, Daniela Toniolo, Giovanni Tonon

**Affiliations:** ^1^Center for Omics Sciences, IRCCS San Raffaele Scientific Institute, Milan, Italy; ^2^Functional Genomics of Cancer Unit, Experimental Oncology Division, IRCCS San Raffaele Scientific Institute, Milan, Italy

**Keywords:** personalized medicine, genomics, sequencing, participatory medicine, GDPR

## Abstract

The emergence of data coming from different venues, as several “omic” approaches, is providing already compelling evidence that the smart use of this information could provide invaluable information to prevent, diagnose and treat human diseases. However, the most daunting challenges remain ahead, as the explosive accumulation of data from additional perspectives, including social graphs, biosensors, and imaging, promise to deliver crucial information that could be exploited for the improvement of the entire human race, both in developed, and developing countries, optimizing health expenses and reaching also the less fortunate sections of the societies. And yet, formidable challenges remain, that pertain for the most part to the collection of the data, their organization, and most relevantly their integration. Here we provide few, pointed examples to the present relevance of these big data approaches in human health as well potential road maps toward the implementation of broader data collections and analyses.

## Introduction

As stated by the World Health Organization (WHO), health represents a “State of complete physical, mental, and social well-being, and not merely the absence of disease or infirmity.” Notwithstanding the challenges, it has been argued that humanity has never been so healthy, safe, knowledgeable, prosperous and ultimately happy (1). Without necessarily endorsing a too positivistic perspective, it appears indeed true that the technological innovations introduced into the medical practice in the past century or so have profoundly improved the outcome for individuals, at least, but not only, in the civilized countries. The introduction of antibiotics, but also the ability to treat a vast array of ailments have improved not only life expectancy, but also the quality of life. We are now entering in another era, which will be centered upon Big Data and that has been heralded, possibly with some exaggeration, revolutionary. We posit that this new perspective is endowed with unique opportunities, but also with menacing threats, which need to be promptly addressed, provided also the unparalleled intrusive capacity of new technologies.

## The All-Encompassing Genomic Medicine?

Genomics seems destined to acquire a central role toward the widespread implementation of the personalized medicine revolution (2, 3). This new framework posits that a Prewomb-to-tomb Assessment is advisable and should be pursued (2). Indeed, the ENCODE initiative has dispelled the widespread perception that most of our DNA is “junk” (4), as genes account for only a minimal portion of its entire sequence. In fact, it now appears that much of the genome is close to regulatory features, as 95% of it lies within 8 kilobases (kb) of a DNA–protein interaction site (as assayed by bound ChIP-seq motifs or DNase I footprints), and 99% is within 1.7 kb of at least one of the biochemical events measured by ENCODE (5). Single nucleotide polymorphisms (SNPs) associated with disease by GWAS studies are enriched within non-coding functional elements, with a majority residing in or near ENCODE-defined regions that are outside of protein-coding genes. This reliance on DNA has been recently fostered by the development of genome-wide polygenic scores for five common diseases, including coronary artery disease, atrial fibrillation, type 2 diabetes, inflammatory bowel disease, and breast cancer (6). These polygenic risk predictors included the assessment, for each condition, of up to 7 million variants, a far cry from the conventional monogenic approaches. These polygenic risk scores could identify a substantially larger fraction of the population than is found by rare monogenic mutations, at comparable or greater disease risk. The recent introduction of next generation sequencing capabilities allowing the comprehensive assessment of the entire genome sequence with few hundred dollars is feeding upon this perspective.

And yet, the assessment of the DNA is most likely not enough to obtain a really comprehensive perspective of the individual, to be exploited to improve health and well-being (2, 7). Other realms ought to be explored, including the transcriptome, the metabolome, the proteome, the microbiome, and the epigenome. There are indeed challenges in exploring these additional metrics, when compared with the assessment of the DNA. They are often less developed from a technological standpoint, in general less stable and often more difficult to measure. Moreover, the most daunting challenge, as yet to be properly addressed, is their integration (see below).

## Beyond the Genome

Beside the collection and the analysis of biological data, other data sets are entering into the arena of personalized medicine. Up to this point, they have not provided robust metrics to discern the feature of individuals within populations, nor to cater reliable predictive markers, and yet there is a widespread enthusiasm and thrust, that their implementation would significantly improve the management of patients and more broadly of the individual health status. A case in point is represented by the exposome, whose goal is to collect a vast array of data, deemed crucial for the well-being, which include diet, pollution, and stress, among others (8). While the collection of these data may appear (and still is) ephemeral, the introduction of new technologies, including applications in smart phones and portable devices, is promising a more standardized, robust mean to record, organize and track the various information included in this realm.

Another approach that holds great promise is the integration of imaging with other data, including genetic information. In the context of the UK Biobank (9), to determine the “genetic architecture” of brain structure and function, Elliott et al. (10) have completed genome-wide association studies of thousands functional and structural brain imaging phenotypes from UK Biobank, from close to more than 8,000 patients. Strikingly, they have found that several of these phenotypes were heritable. Notably, they also detected clusters of associations between single nucleotide polymorphisms and imaging phenotypes. Moreover, an association between these imaging patterns and the future development of neurological syndromes was found.

Collecting cognitive information, albeit sensitive from the protection of the personal data perspective, remains a largely untapped, and yet promising resource. As an example, again UK biobank has been prospectively collecting these data, through battery of tests, designed to be administered in a short time frame (roughly 15 min), without the need of an examiner, since digitals are used to collect the data. Five cognitive abilities are assessed, namely reasoning (ability to solve verbal and numeric reasoning problems), reaction time (response time to visual stimuli), numeric, visuo-spatial and prospective memory (11). Indeed, from a clinical perspective, cognitive abilities are an important component in epidemiologic research, as cognitive impairment is a risk factor for a broad range of health-related conditions, including cardiovascular diseases and earlier mortality. From a methodological standpoint, the measurements to provide a score of cognitive ability is complex, since the different cognitive scores are inter-correlated. For this reason methodologies based on Principal Components Analysis (PCA) are used to highlight the major sources of variance in a “general factor of cognitive ability” (11).

## Challenges of Data Integration

Despite the need has existed for a long time, and the realization that effective translation from research to cure will require systematic access and integration of research and health care at a large scale and possibly across institutions and countries, reliable tools to integrate data sets remains one of the most daunting challenges faced by the field. Even the combination in a unique model of omic data is fraught with controversies, and lacks a consensual, robust methodology.

Approaches such as Non-negative Matrix Factorization (NMF) and more recently Multi Omics Factor Analysis (MOFA) have gained traction, and appear to provide at last inroads toward this goal. NMF was first proposed as a method to decompose images, for example, faces into parts reminiscent of features such as eyes, nose, mouth, cheeks, and chin. It has been then applied to microarray data, where it was able to reduce the dimension of expression data from thousands of genes to few metagenes (12). We then applied NMF for the first time to the analysis of DNA copy number variation data (13). Lately, NMF has been used to integrate data from different sources, e.g., single cell RNA and single cell ATAC-seq data (14). Another promising tool to integrate data is Multi-Omics Factor Analysis (MOFA), which aims to infer hidden factors underlying biological and technical sources of variability. To this end, MOFA defines axes of heterogeneity, either shared or specific across data modalities (15).

On more general terms, one of the main obstacles toward data integration is their comparability and consistency. Biomedical data are oftentimes heterogeneous, incomplete and imprecise by nature. Even the task of obtaining and integrating Electronic Health Records (EHRs) across hospitals, within a country, has proven much more complex than anticipated, even in the most advanced health care systems (16). Indeed, even in the US where more than 90% of the hospitals have adopted EHRs tools, these are obtained from various companies, and their ability to communicate remain limited. In Europe as well, initiatives are ongoing to establish robust platforms for collecting and sharing standardized data, such as DIFUTURE in Germany (17) and other similar initiatives in single EU states, as Alleanza contro il Cancro in Italy (18). When compared with the US, one advantage in Europe seems to be the possibility to generate networks like Data Integration Centers that could collect and process data at the national and supranational level.

The introduction of machine learning within the frame of Artificial Intelligence (AI) approaches appears particularly suited to address these challenges, although even within this realm quantity of the original data and their proper standardization remain of paramount importance (19, 20). Also, at several levels that go beyond the obvious privacy concerns, AI poses serious concerns, including adversarial attacks (21), hence appropriate ethical boundaries would need to be implemented (22).

## Ethical Challenges, the GDPR, and Beyond

The availability of “big data” is posing significant challenges also from an ethical standpoint. The recent introduction in the European community of the EU General Data Protection Regulation (GDPR) is a comprehensive attempt to protect privacy rights of the individuals, while fostering research and more specifically free scientific data exchange (23). Despite its considerable sanctionatory harshness (up to 4% of a company's yearly global revenues, in case of non-compliance), the general philosophy underlying the GDPR revolves around decentralization, through the delegation of responsibility to data controllers. Additionally, the GDPR increases the role of internal review boards (IRBs) and ethical committees, with an enhanced role in policy making. Albeit it is too early to properly assess the impact and the role of GDPR in the management of Big Data, nevertheless it is certain that tensions will arise around the management of the data and to properly regulate their use and who could assess them. Two issues in particular that are emerging have to do to the need to request again to patients whether their data could be used, for research projects that may go beyond the specific scope of the initial consent, and whether the somehow relaxed rules imposed on academia in handling data could be also extended to commercial players under the provision that the data have been sought and obtained for “scientific research.”

Along these lines, start-up genetics companies are now offering genome sequencing at no cost. Even if individual data will remain anonymous and under the informed consent approval, this approach may foresee the commercialization of genetic information. Other companies have set up platforms that connect people and commercial entities to buy and sell DNA, sequencing, health-related information.

As it has been claimed, the GDPR, if loosely interpreted, may lead to the indefinite storage of personal, sensitive data, including genetic, also by commercial research entities, for any research purposes, and even processed without the data subject knowledge. In fact, within the frame of this interpretation of the GDPR, the individual may not have even the option to “opt-out” (24). In fact, the exploitation of genealogy databases, or more broadly consumer genomics databases, allows to identify up to 60%, and soon nearly any US-individual of European- descent in the near future, using demographic identifiers, including research participants of public sequencing projects (25). These approaches have been recently successfully used by law enforcement agencies to identify criminals, posing significant ethical and legal challenges (26).

## The Future: Participatory Medicine

As noted above, the new era of Big data in medicine provides several new challenges, alongside great opportunities, to improve the health for human kind, not only for wealthy nations, but also for underdeveloped countries. To this end, it is fair to say that a profound cultural shift ought to occur, which entails professional figures and stakeholders that up to now have not been engaged in previous revolutions. Patients, doctors, but also clinical laboratory technicians and researchers would need to acquire new knowledge, and most relevantly interact and acquire novel frames of mind and perspectives, leading to an entirely overhauled health eco-system (27). Clinicians would have to engage and interact more pervasively with clinical laboratory technicians and researchers, and researchers and clinical laboratory technicians to work more closely together. Also, patients would be required to acquire notions of genetics, with the final goal being the removal of barriers that at the present time are still preventing the delivery of the best treatments to patients, to arrive to a form of “participatory” medicine among patients, doctors and their community (28). Along this line, the whole framework of data, information, knowledge, and wisdom (DIKW) has been proposed for personalized medicine, whereby “smart patients” may take a primary and leading role in their healthcare, assuming higher levels of responsibility for their own health and wellness (29).

We hope that this goal is not over-ambitious and could be reached in a future not too far.

## Author Contributions

All authors listed have made a substantial, direct and intellectual contribution to the work, and approved it for publication.

### Conflict of Interest

The authors declare that the research was conducted in the absence of any commercial or financial relationships that could be construed as a potential conflict of interest.
